# *Mycobacterium avium* inhibits protein kinase C and MARCKS phosphorylation in human cystic fibrosis and non-cystic fibrosis cells

**DOI:** 10.1371/journal.pone.0308299

**Published:** 2024-10-16

**Authors:** Kevin J. Kokesh, Niharika Bala, Yunus E. Dogan, Van-Anh L. Nguyen, Marcus Costa, Abdel Alli

**Affiliations:** 1 Department of Pediatrics, University of Florida College of Medicine, Gainesville, Florida, United States of America; 2 Department of Physiology and Aging, University of Florida College of Medicine, Gainesville, Florida, United States of America; 3 Department of Pediatrics, Erciyes University of Medicine, Kayseri, Turkey; 4 Department of Medicine, Division of Division of Nephrology, Hypertension, and Renal Transplantation, University of Florida College of Medicine, Gainesville, Florida, United States of America; University of Nebraska Medical Center, UNITED STATES OF AMERICA

## Abstract

In cystic fibrosis (CF), there is abnormal translocation and function of the cystic fibrosis transmembrane conductance regulator (CFTR) and an upregulation of the epithelial sodium channel (ENaC). This leads to hyperabsorption of sodium and fluid from the airway, dehydrated mucus, and an increased risk of respiratory infections. In this study, we performed a proteomic assessment of differentially regulated proteins from CF and non-CF small airway epithelial cells (SAEC) that are sensitive to *Mycobacterium avium*. CF SAEC and normal non-CF SAEC were infected with *M*. *avium* before the cells were harvested for protein. Protein kinase C (PKC) activity was greater in the CF cells compared to the non-CF cells, but the activity was significantly attenuated in both cell types after infection with *M*. *avium* compared to vehicle. Western blot and densitometric analysis showed a significant increase in cathepsin B protein expression in *M*. *avium* infected CF cells. Myristoylated alanine rich C-kinase substrate (MARCKS) protein was one of several differentially expressed proteins between the groups that was identified by mass spectrometry-based proteomics. Total MARCKS protein expression was greater in CF cells compared to non-CF cells. Phosphorylation of MARCKS at serine 163 was also greater in CF cells compared to non-CF cells after treating both groups of cells with *M*. *avium*. Taken together, MARCKS protein is upregulated in CF cells and there is decreased phosphorylation of the protein due to a decrease in PKC activity and presumably increased cathepsin B mediated proteolysis of the protein after *M*. *avium* infection.

## Introduction

Cystic fibrosis (CF) is among the most common genetic diseases, affecting approximately 1 in 3000 Caucasian births in North America and Europe [1]. CF primarily affects cystic fibrosis transmembrane conductance regulator (CFTR) channels via disease-causing mutations in the CFTR gene, of which more than 2000 have been identified to date [2]. The gene encodes the CFTR protein, which conducts chloride and bicarbonate across epithelial cell membranes in the lungs, upper airways, exocrine pancreas, and gut. The appropriate exchange of ions is critical in maintaining homeostatic surface conditions, namely hydration and acidity, at the epithelial surfaces of these organs. Dysregulation in the CFTR channel therefore leads to mucociliary clearance impairment within the airways, predisposing the CF patient to frequent and recurrent infections, many of which become resistant to typical antimicrobial treatments. While most of the infection in CF patients is secondary to so-called “typical” pathogenic bacteria such as *Staphylococcus aureus* and *Pseudomonas aeruginosa*, increasing importance is now being attributed to a class of atypical bacteria known as non-tuberculous mycobacteria (NTM).

NTM are a group of important pathogenic bacteria that are now known to cause serious morbidity in cystic fibrosis patients [3]. These bacteria are commonly found in soil and water; however, NTM are also present in animals, water, and indoor plumbing [4–6]. Within the CF population, NTM colonization prevalence has been estimated by various studies at 3 to 23% [3], but not all patients with NTM-positive cultures have clinical manifestations of disease or require treatment [7]. The inhomogeneous geographic distribution of NTMs additionally contributes to their complex pathology. Although they are ubiquitous worldwide, the species incidence varies widely between European countries, the United States, and the rest of the world, as well as within individual countries [8–12]. This heterogeneity has ramifications on diagnosis and treatment approach, and as such there is neither a singular diagnostic “identification” algorithm [9] nor a standard treatment plan. In addition, the overwhelming bulk of investigations of NTM within the CF population have been descriptive at the population level (i.e., defining prevalence and risk factors) [7, 11], and have highlighted the knowledge gap in this area of pulmonary medicine. In 2016, to address these issues, the Cystic Fibrosis Foundation (CFF) published a set of clinical care guidelines based on a review of contemporary literature [13]. Despite these efforts, NTM infection in CF patients is still largely managed on a case-by-case basis and more research is needed to understand its complex pathogenesis and interaction with the host immune response.

Emerging evidence indicates proteases play an important role in the progression of cystic fibrosis. Cathepsins are proteases primarily found in acidic endosomal and lysosomal compartments, where they have functions in energy metabolism, intracellular degradation of proteins, and play a critical role in the host immune response [14]. The presence of cathepsin B at the apical membrane of bronchial epithelia in both normal and CF donor lungs has been demonstrated [15]. Furthermore, cathepsin B has also been shown to be enriched in extracellular vesicles [16], and is known to play a role in the regulation of epithelial transports mechanisms in pathophysiology [17, 18]. There is also evidence of increased cathepsin B activity in animal model alveolar macrophages [19]. In the respiratory tract, cathepsin B has been linked to extracellular matrix remodeling in addition to regulation of the immune response [20]. Cathepsins are typically made as pre-proteins and auto-cleavage to active proteases occurs at acidic pH [20]. This has made the cathepsin family of proteins an area of focus in CF research, as the air surface liquid (ASL) is acidic in CF patients due to a reduction in physiologic CFTR-mediated bicarbonate secretion [21]; this acidity has the doubly problematic effect of inhibiting bacterial killing per se, via downregulation of cationic antimicrobial peptides [22], as well as excessively activating proteases such as neutrophil elastase and cathepsin proteases [23]. In combination, these effects promote airways’ bacterial colonization and the pro-inflammatory state seen in CF patients [23]. Cathepsin G, for instance, has been implicated in mucus secretion [24, 25]. Cathepsins have been shown to mediate neutrophilic phagocytosis of some microbes such as *Aspergillus fumigatus* [24] and *Staphylococcus aureus* [26].

Relatively few studies to date have investigated the effects of NTM infection on cathepsin expression and function, and to our knowledge no prior study has evaluated this relationship in cystic fibrosis patients. However, previous research with *M*. *tuberculosis* has suggested that expression of cathepsins B, S and L is down-regulated in TB-infected cells, an effect that was not seen in a non-pathogenic mycobacterial species, *M*. *smegmatis* [27]. Directly targeting cathepsin expression upon infection of activated macrophages resulted in the upregulation of multiple cathepsins, suggesting that the bacteria has evolved techniques to decrease cathepsin activity [27]. Therefore, it has been hypothesized that mycobacterial suppression of cathepsins’ activity and therefore, lysosomal killing may be responsible for the bacteria’s robust intracellular survival advantage [27]. Thus far, pathogenic mycobacteria have been shown to reduce the activity of cathepsins sourced from monocytes in vitro [28]. In the present study, we assessed whether and to what extent there is a difference in cathepsin expression and activity between NTM-infected epithelial cells from CF patients and non-CF subjects.

## Materials and methods

### Cell culture

Small airway epithelial cell (SAEC) (CC-2547) and diseased small airway epithelial cells (CC-2933) were purchased from Lonza (Walkersville, MD) and maintained in Small Airway Epithelial Basal Medium (CC-3119) supplemented with SAGM SingleQuot Kit Suppl. & Growth Factors (CC-4124) from Lonza. The media was exchanged every 3 days and only cells with a passage number of less than 5 were used for experiments.

### Mycobacterium avium

*M*. *avium* serotype 2 (Cat. No. #0801663) was purchased from ZeptoMetrix (Buffalo, NY). CF and non-CF cells were infected with different multiplicities of infection (MOI) in complete growth media for 8 hours.

### SDS-PAGE and Western blotting

SDS-PAGE and Western blotting were performed as previously described by our group [29] with the following modifications. A Criterion electrophoresis system (BioRad; Hercule, CA, USA) was used to resolve total protein from SAEC and DSAEC lysates for one hour at 200 V. The proteins were then transferred to nitrocellulose membranes (ThermoFisher Scientific) for 1 h at 100 V in chilled Towbin Buffer (20% methanol, 25 mM Tris, 192 mM glycine). Ponceau staining was performed to assess lane loading. The membranes were blocked in a 5% non-fat milk 1xTris Buffered Saline (TBS) solution at room temperature for 1 h. The membranes were washed with 1xTBS solution before being incubated at 4°C for 8–12 h with a 1:1000 dilution of primary antibody (Table 1) solution prepared in 5% BSA 1XTBS. The membranes were washed with 1X TBS and then incubated at room temperature for 1 h with a 1:3000 dilution of anti-rabbit or anti-mouse secondary antibody (BioRad) prepared in blocking solution. The membranes were washed with 1X TBS, and then incubated with enhanced chemiluminescence (ECL) solution (ThermoFisher Scientific) before being imaged on a ChemiDoc imager (BioRad).

**Table 1:** Antibodies used in this study.

Antibody	Application	Company	Catalog Number
Cathepsin B	Western blotting	Cell Signaling Technology	31718S
MARCKS	Immunofluorescence	Santa Cruz	sc100777
MARCKS	Western blotting	Abcam	ab72459
Phospho MARCKS (ser 163)	Western blotting, Immunofluorescence	Invitrogen	PA5-36852
Alexa Fluor 488	Immunofluorescence	Invitrogen	A21206
Alexa Fluor 568	Immunofluorescence	Invitrogen	A10037

### PKC activity assays

The activity of PKC was measured while following instructions from the manufacturer (abcam Ab139437). A plate reader (Tecan) equipped with Magellan Software was used to read the plates.

### Proteomics

For in-solution digestion, SAEC and DSAEC cells were lysed using an EasyPep^™^ MS Sample Prep Kit (Thermo Fisher Scientific). A Qubit was used to determine total protein concentration and calculate the specific volume of each sample to have 20 mg of total protein for the digestion as previously described by Dill et al. [30] and Chao et al. [31], with the following modifications. A sequencing grade trypsin/lys C rapid digestion kit (Promega; Madison WI) was used to digest each sample while following the instructions from the manufacturer. A 1:3 volume ratio of sample to rapid digestion buffer was used before the sample was incubated for 30 minutes at 56°C with 1 ml of dithiothreitol solution (0.1 M in 100 mM ammonium bicarbonate). Next, 0.54 mL of 55 mM Iodoacetamide prepared in 100 mM ammonium bicarbonate was added and incubated in the dark at room temperature for 30 minutes. Afterwards, 1 ml of the freshly prepared enzyme was added to the samples before being incubated for 1 h at 70°C. The addition of 0.5% TFA was used to stop the digestion. To minimize non-specific cleavage with high quality tryptic peptides, the MS analysis was performed immediately after the digestion reaction. The Nano-liquid chromatography tandem mass spectrometer system consisted of a Thermo Scientific Q Exactive HF Orbitrap mass spectrometer that was equipped with an EASY Spray nanospray source (Thermo Scientific) which was run in positive ion mode using an UltiMate^™^ 3000 RSLCnano system (Thermo Scientific). The mobile phase A consisted of water containing 0.1% formic acid, while mobile phase B consisted of acetonitrile with 0.1% formic acid. For the loading pump, the mobile phase A consisted of water containing 0.1% trifluoracetic acid. A PharmaFluidics mPACä C18 trapping column (C18, 2.5 mm inter-pillar distance, 5 mm pillar diameter, and 10 mm length) was injected with 5 mL of sample at 10 mL/ml flow rate was after being held for 3 minutes and washed with 1%B to desalt and concentrate the peptides. The peptides were eluted off the trap onto the column. Chromatographic separations (C18, 5 mm pillar diameter, 2.5 mm inter-pillar distance, 50 cm length) were achieved using PharmaFluidics 50 cm mPAC ä A temperature of 40°C was maintained for the column and a flowrate of 750 nL 1/min was used for the first 15 min before the flow was reduced to 300 nL 1/min. A gradient of 1% B to 20%B over 100 minutes and then 45% B in 20 minutes for a total run-time of 150 minutes (Table 2) was used to directly elute the peptides off the column into the Q Exactive system.

**Table 2:** Time and flowrate used for proteomics.

Time (min)	% B	Flow Rate (nL/min)
0	1	750
3	1	750
15	5	750
15.1	5	300
100	20	300
123	45	300
130	95	300
135	95	300
135.1	1	300
150	1	300

The EASY Spray source was used with a capillary temperature of 200°C and a spray voltage of 1.5 kV. A full scan analysis was recorded between 375–1575 Da at a 60,000 resolution, with an MS/MS scan at a 15,000 resolution to obtain product ion spectra and determine amino acid sequence in consecutive instrument scans of the fifteen most abundant peaks in the spectrum. Next, 3e6 ions for full scan (maximum ion injection time of 50 ms) were used for the AGC Target ion number while 2e5 ions was used for MS2 mode (55 ms). For both the full scan and MS2 scan, the Micro scan number was set to 1. The HCD fragmentation energy (N)CE/stepped NCE was set to 28 with a 4 m/z isolation window. Singly charged ions were omitted from the MS2 scan. Dynamic exclusion consisting of a repeat count of 1 within 15 seconds was utilized to exclude isotopes. An internal lock mass consisted of a Siloxane background peak at 445.12003. The integrity and performance of the column and instrument was evaluated using HeLa protein digest standard.

Sequest (Thermo Fisher Scientific, San Jose, CA, USA; version IseNode in Proteome Discoverer 3.0.1.27) was used to analyze the MS/MS spectra. Sequest was utilized to search Homo sapiens (NcbiAV TaxID = 9606) (v2023-01-24) with trypsin being the digestion enzyme. The Sequest searches utilized a parent ion tolerance of 10.0 ppm and a fragment ion mass tolerance of 0.020 Da. Carbamidomethyl of cysteine was considered a fixed modification while variable modifications included Met-loss of methionine, met-loss+Acetyl of methionine, oxidation of methionine and acetyl of the n-terminus. FunRich analysis was used to map out pathway analysis using the proteomics database.

Proteome Discoverer (Thermo Fisher Scientific vs 2.4.0.305) was used for Precursor ion intensity label free quantitation. A comparison was made between SAEC and DSAEC cells infected with vehicle or *M*. *avium*, utilizing a “non-nested” study factor. Normalization was derived by using all peptides. The abundance of protein was calculated using summed abundances, while considering the mean of the protein abundances calculated by summing sample abundances of the connected peptide groups. P-values were calculated using Fisher’s exact test (pairwise ratio-based) while including a low intensity resampling value. Benjamini-Hochberg. Statistical Analysis was used to calculate the adjusted p-values.

### Fluorescence microscopy of phospho-MARCKS expression in non-CF and CF cells

CF and non-CF cells were grown to 20 percent confluency in 35mm glass bottom cell culture dishes (MatTek, Ashland MA). The cells were fixed for 10 minutes at -20°C in an ice cold 1:1 ratio of methanol/acetone solution (v/v). Next, the cells were incubated in blocking solution (2.5% normal horse serum) for 20 minutes at room temperature followed by incubation with primary antibodies (mouse monoclonal anti-MARCKS antibody and rabbit polyclonal anti-phospho-MARCKS ser 163) prepared at a dilution of 1:200 in blocking solution for 45 minutes at room temperature. After a series of two washes with 1XPBS, the cells were incubated with a 1:200 dilution of conjugated secondary antibodies (Table 1) for 30 minutes at room temperature. After a series of two washes with 1XPBS, antifade mounting media containing DAPI (Vector labs) was added, and the cells were cover slipped and imaged on a fluorescence microscope (Olympus) using a 40X objective.

## Results

### PKC is down-regulated in human CF and non-CF cells infected with *M*. *avium*

**Fig 1:**
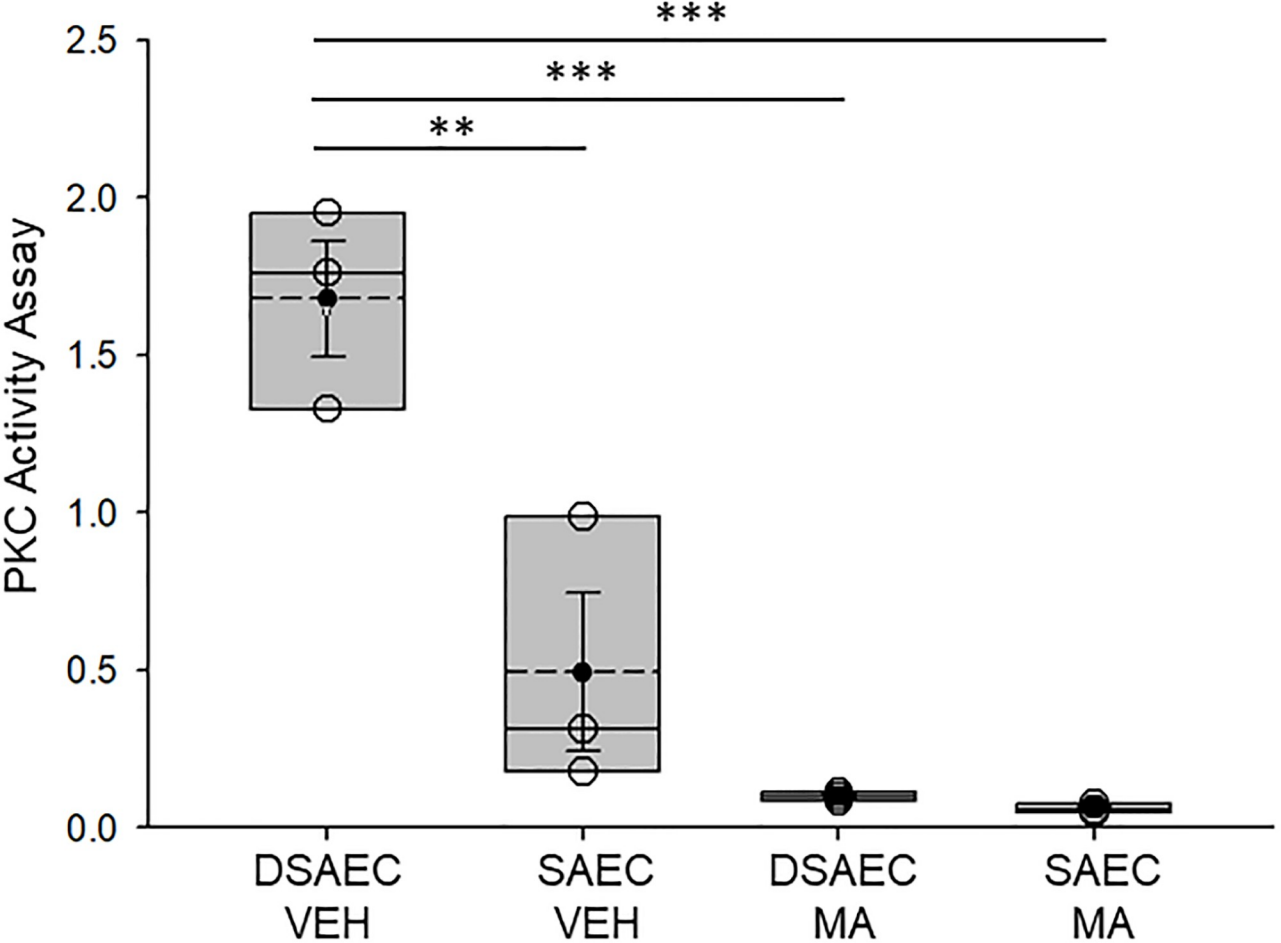
Protein kinase C (PKC) activity in CF and non-CF cells infected with *M*. *avium* or vehicle. DSAEC refers to CF small airway epithelial cells. SAEC refers to non-CF small airway epithelial cells. MA refers to *M*. *avium* infection. VEH refers to vehicle infection. SAEC refers to non-CF small airway epithelial cells and DAEC refers to CF small airway epithelial cells. N = 3 independent experiments per group. ** p value <0.01, *** p value < 0.001.

Since PKC is known to directly activate CFTR-mediated chloride transport [32], we aimed to determine whether NTM infection affects PKC activity in human CF and non-CF small airway epithelial cells. Cells were infected with *M*. *avium* (MOI 10) or vehicle for 8 hours and then PKC activity was measured in the different groups. As shown in Fig 1, basal PKC activity in the non-CF SAEC was significantly lower than that CF SAEC. Additionally, PKC activity was significantly reduced in both non-CF and CF cells infected with *M*. *avium* compared to vehicle (Fig 1).

### Cathepsin B is augmented in human CF and non-CF small airway epithelial cells infected with *M*. *avium*

**Fig 2:**
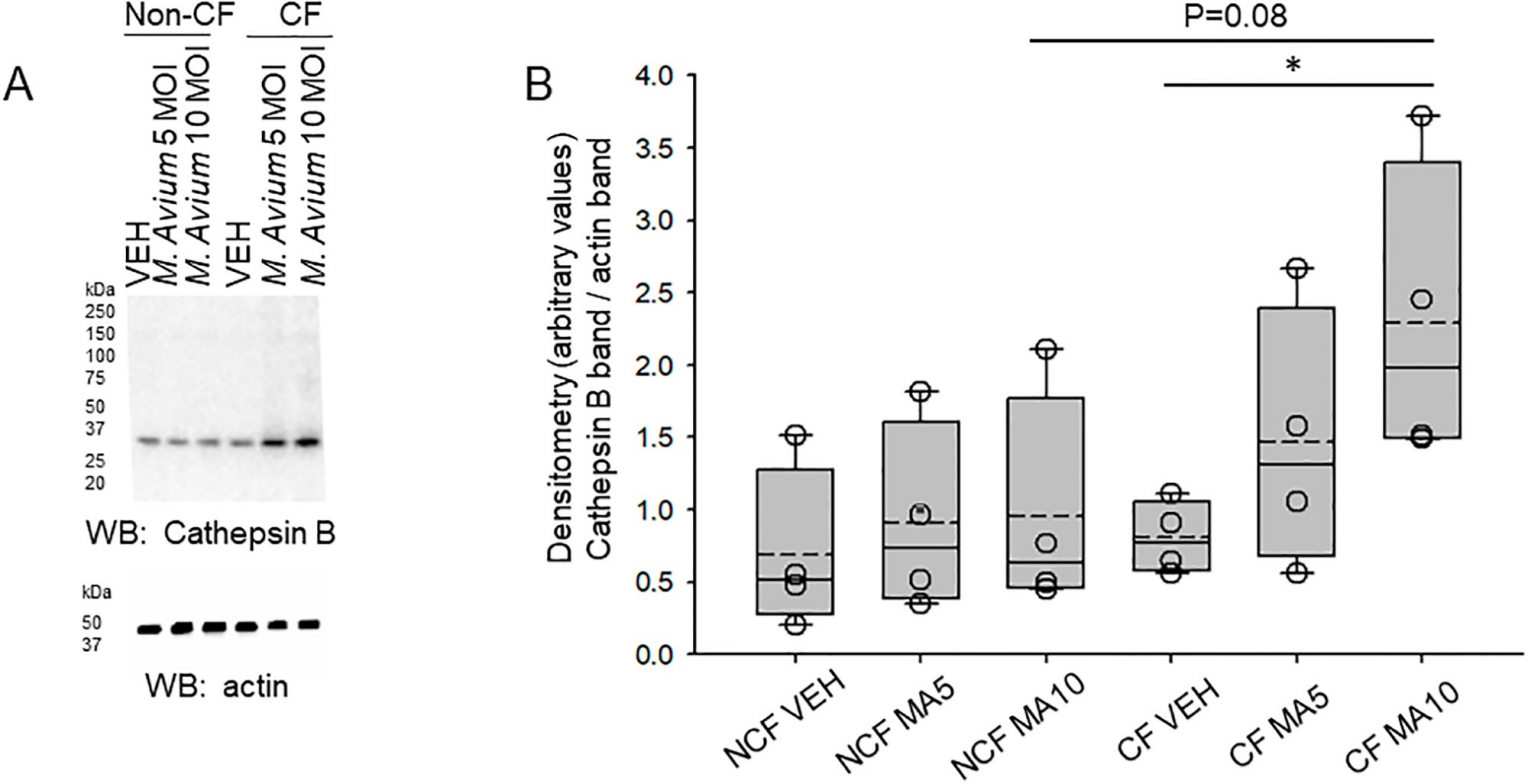
Cathepsin B protein expression in non-CF and CF cells infected with vehicle or *M*. *avium*. Non-CF and CF cells were infected with *M*. *avium* with the indicated MOI for 8 hours. A. Representative Western blot of cathepsin B protein expression (top). Western blot of actin (bottom) to assess lane loading. B. Densitometric analysis of the cathepsin B band normalized to the actin band in panel A. VEH refers to vehicle treatment. SAEC refers to non-CF small airway epithelial cells and DAEC refers to CF small airway epithelial cells. MOI refers to multiplicities of infection. N = 3 independent experiments per group. * represents a p-value<0.05.

Prior studies have shown cathepsin S is upregulated in CF cells [33, 34] and cathepsin B has been shown to play a role in the pathophysiology of CF lung disease [20]. Here we investigated changes in cathepsin B protein expression between CF and non-CF small airway epithelial cells, and whether *M*. *avium* infection affects the expression of cathepsin B protein in the two cell types. As shown in Fig 2, there was greater cathepsin B protein expression in the CF cells after infection with MA.

### Proteomic analysis of non-CF and CF cells infected with vehicle or *M*. *avium*

**Fig 3:**
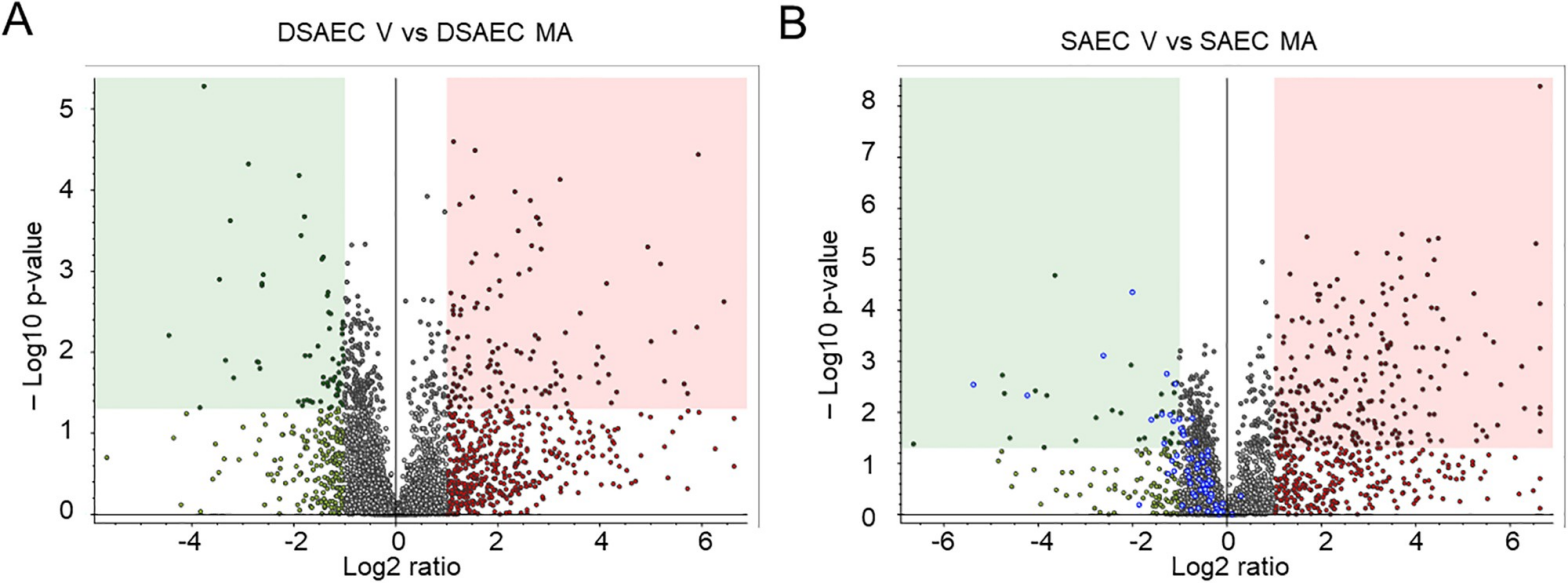
Volcano plot of differentially expressed proteins in CF and non-CF cells infected with vehicle or *M*. *avium*. The volcano plot illustrates differentially expressed proteins (DEP) between the DSAEC (CF small airway epithelial cells) and SAEC (non-CF small airway epithelial cells) infected with vehicle (V) or *M*. *avium (MA)* (MOI 10) for 8 hours. The green dots represent down-regulated proteins, red dots represent up-regulated proteins, and gray dots show proteins with no statistically significant difference. MOI refers to multiplicities of infection.

**Fig 4:**
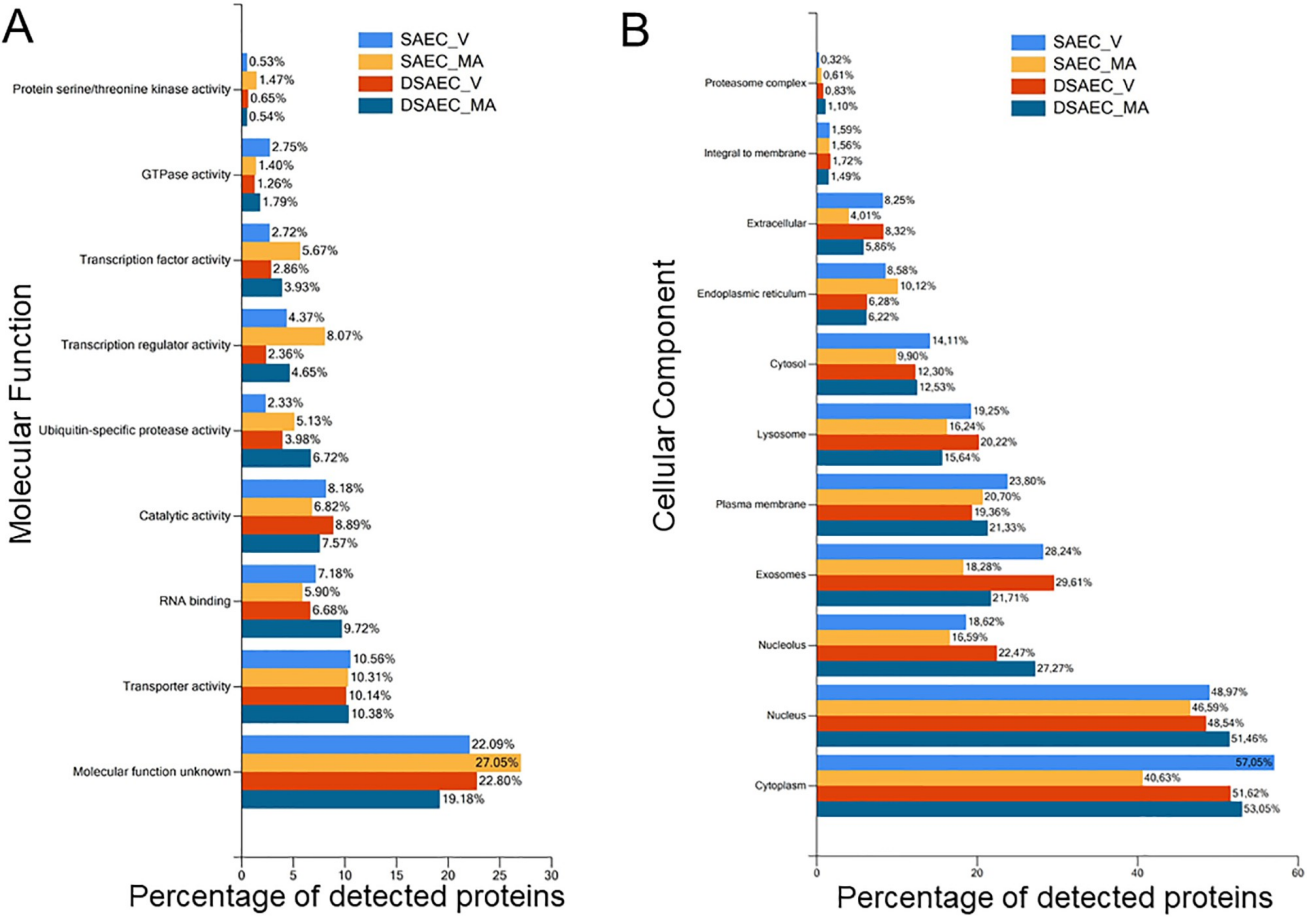
Molecular function and cellular component analysis of differentially expressed proteins in CF and non-CF cells infected with *M*. *avium* or vehicle. A. Molecular Function. B. Cellular Component. SAEC refers to non-CF small airway epithelial cells and DAEC refers to CF small airway epithelial cells. V refers to vehicle. MA refers to *M*. *avium*.

**Fig 5:**
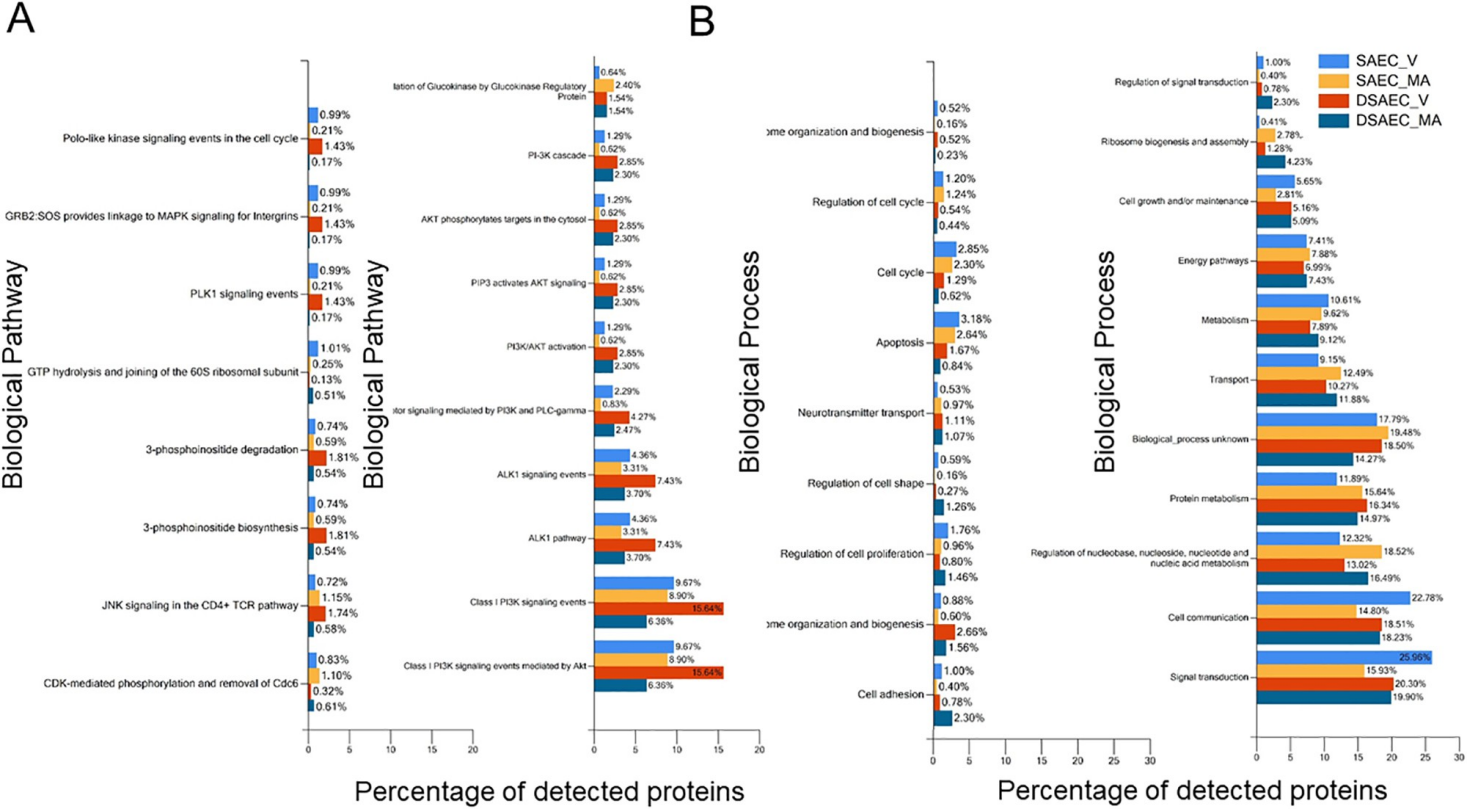
Biological pathway and biological process analysis of differentially expressed proteins in CF and non-CF cells infected with *M*. *avium* or vehicle. A. Biological Pathway. B. Biological Process. SAEC refers to non-CF small airway epithelial cells and DAEC refers to CF small airway epithelial cells. V refers to vehicle. MA refers to *M*. *avium*.

Next, we performed a proteomics analysis to identify putative proteins that could be involved in the mechanism by which *M*. *avium* differentially regulates proteins that associated with either the PKC or the cathepsin pathway. CF and non-CF small airway epithelial cells were infected with *M*. *avium* or vehicle for 8 hours and the cell lysates were then subject to LC-MS/MS based proteomics. Several differentially expressed proteins were identified as shown in the volcano plot in Fig 3. Bioinformatic analysis was performed using the proteomic dataset and the FunRich open-access proteomics software [35] to determine which pathways and cellular functions were most affected between CF and non-CF cells infected with *M*. *avium* compared to vehicle. Our results showed that the most affected molecular functions were transporter activity, RNA binding, catalytic activity, and ubiquitin-specific pathways (Fig 4A). Meanwhile, the cellular components that were most affected between the *M*. *avium* infection and vehicle treatment in the CF and non-CF cells were the cytoplasm, nucleus, nucleolus, exosomes, plasma membrane, and lysosomes (Fig 4B). The FunRich software was also used to map out biological pathways and processes most affected by *M*. *avium*. These analyses revealed that the biological pathways that were most affected between CF and non-CF cells infected with *M*. *avium*, or vehicle were various kinase pathways. These included PI3K, ALK1, AKT, JNK PLK, and CDK (Fig 5).

### MARCKS protein is augmented in CF small airway epithelial cells and after *M*. *avium* infection

**Fig 6:**
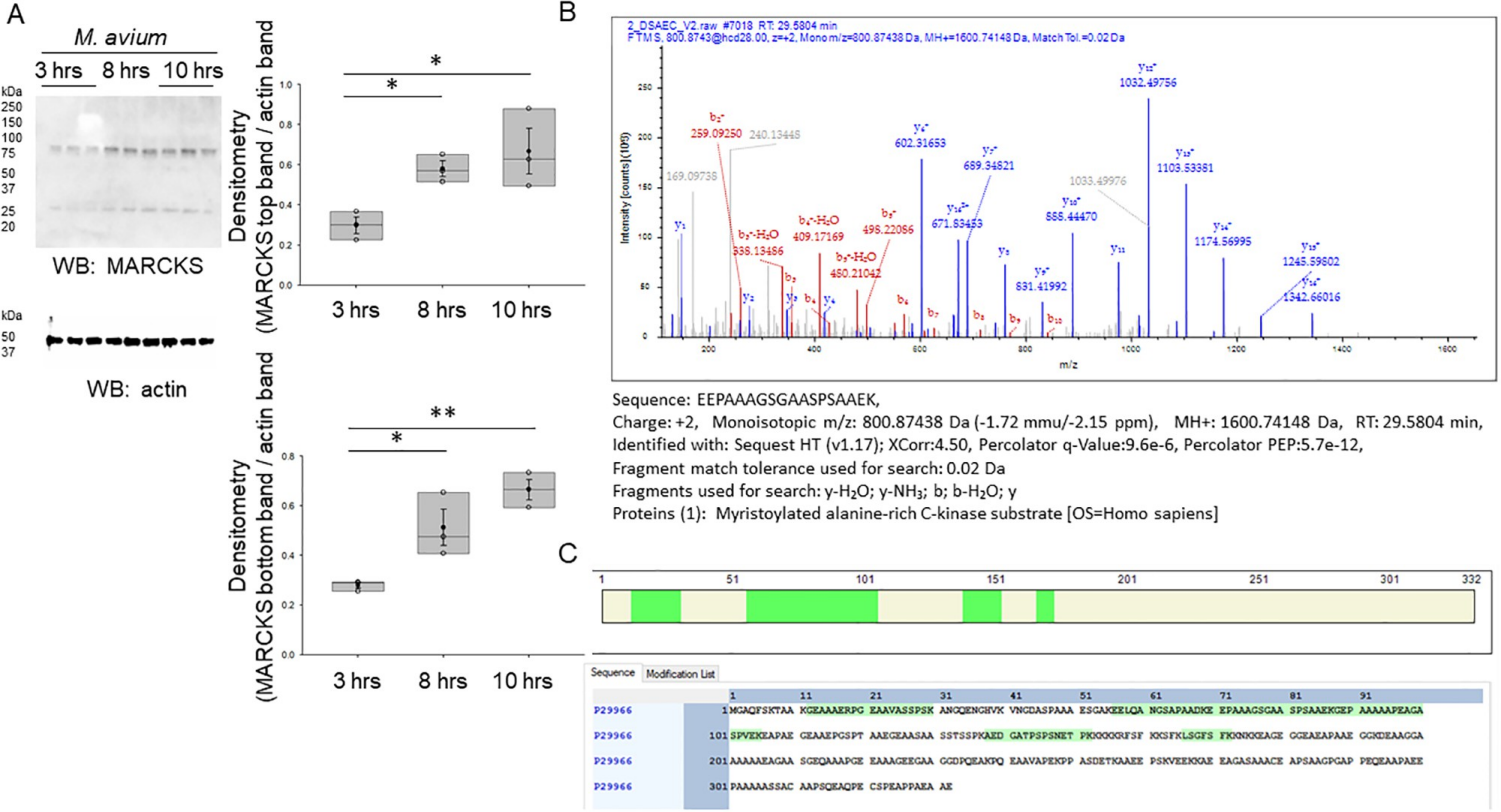
Proteomic and Western blot analysis of MARCKS protein in CF and non-CF cells infected with *M*. *avium* or vehicle. A. Representative Western blot showing total MARCKS protein expression (top) after treating non-CF cells with *Mycobacterium avium* for different periods of time of 3, 5, and 8 hours. Western blot of actin to assess lane loading. Densitometric analysis (bottom) of the Western blot of MARCKS normalized to actin. B. Spectrogram showing the mass-to-charge values (m/z) of MARCKS protein. C. Surrogate peptides corresponding to MARCKS protein. SAEC refers to non-CF small airway epithelial cells and DAEC refers to CF small airway epithelial cells.

Since MARCKS is one of the most prominent substrates of PKC, we investigated whether MARCKS protein expression was affected by *M*. *avium* infection as seen for PKC activity. Western blot and densitometric analysis showed a significant upregulation of MARCKS protein expression in non-CF cells infected with *M*. *avium* (MOI 10) for 8 hours and 10 hours, compared to cells infected with the same dose for 3 hours, but there was no appreciable change between 8 and 10 hours of *M*. *avium* infection (Fig 6A). Therefore, we performed proteomics using CF and non-CF cells infected with *M*. *avium* (10 MOI) for 8 hours. We sought to corroborate the upregulation of MARCKS protein expression by proteomics. The proteomic dataset (https://figshare.com/account/items/24012678/edit) confirmed MARCKS protein was among the upregulated proteins in CF small airway epithelial cells infected with *M*. *avium* compared to vehicle treated cells (Fig 6B and 6C).

### Total MARCKS protein expression is augmented in both non-CF and CF cells infected with *M*. *avium*

**Fig 7:**
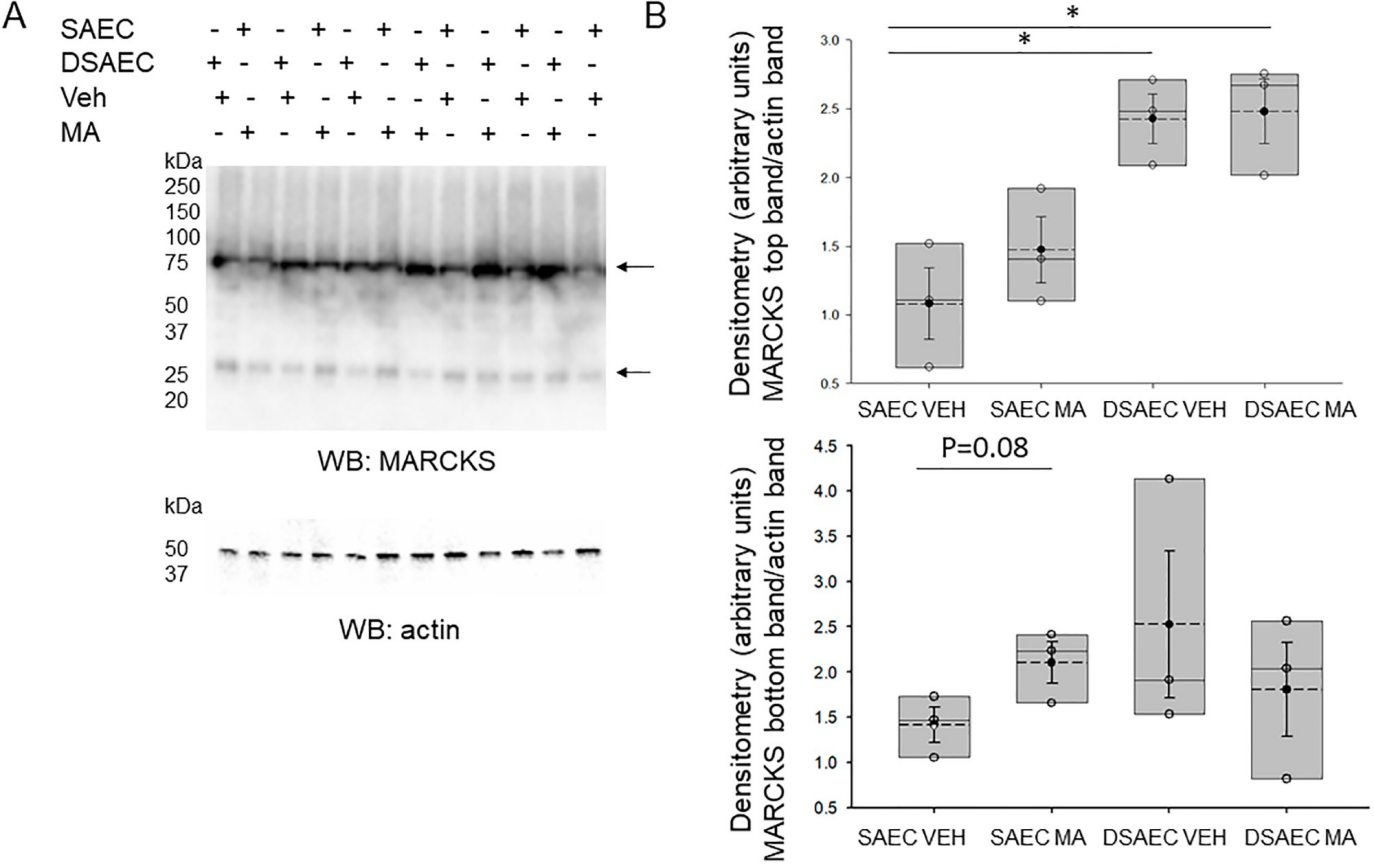
Western blot and densitometric analysis of total MARCKS protein in CF and non-CF cells infected with vehicle or MA. A. Western blot analysis of MARCKS total protein in CF (DSAEC) and non-CF cells (SAEC) infected with *M*. *avium* (MA) or vehicle (Veh) for 8 hours. Western blot of actin to assess lane loading. B. Densitometric analysis of the MARCKS band normalized to the actin band. * indicates a p-value of <0.05.

Like non-CF cells, Western blot and densitometric analysis was performed for total MARCKS protein expression in CF cells with *M*. *avium* (MOI 10) for 8 hours. Although there is an apparent increase in MARCKS protein expression after *M*. *avium* infection compared to vehicle treatment in non-CF cells (Fig 7), the expression of total MARCKS protein in CF cells between the *M*. *avium* infection and vehicle treatment groups were comparable. However, basal levels of total MARCKS protein expression in CF cells infected with vehicle were significantly greater compared to non-CF cells infected with vehicle (Fig 7). Similarly, there was a trend for increased MARCKS protein expression in CF cells infected with *M*. *avium* compared to non-CF cells infected with *M*. *avium* (Fig 7).

### Phosphorylation of serine 163 of MARCKS protein is augmented in CF cells compared to non-CF cells after infection of *M*. *avium*

**Fig 8:**
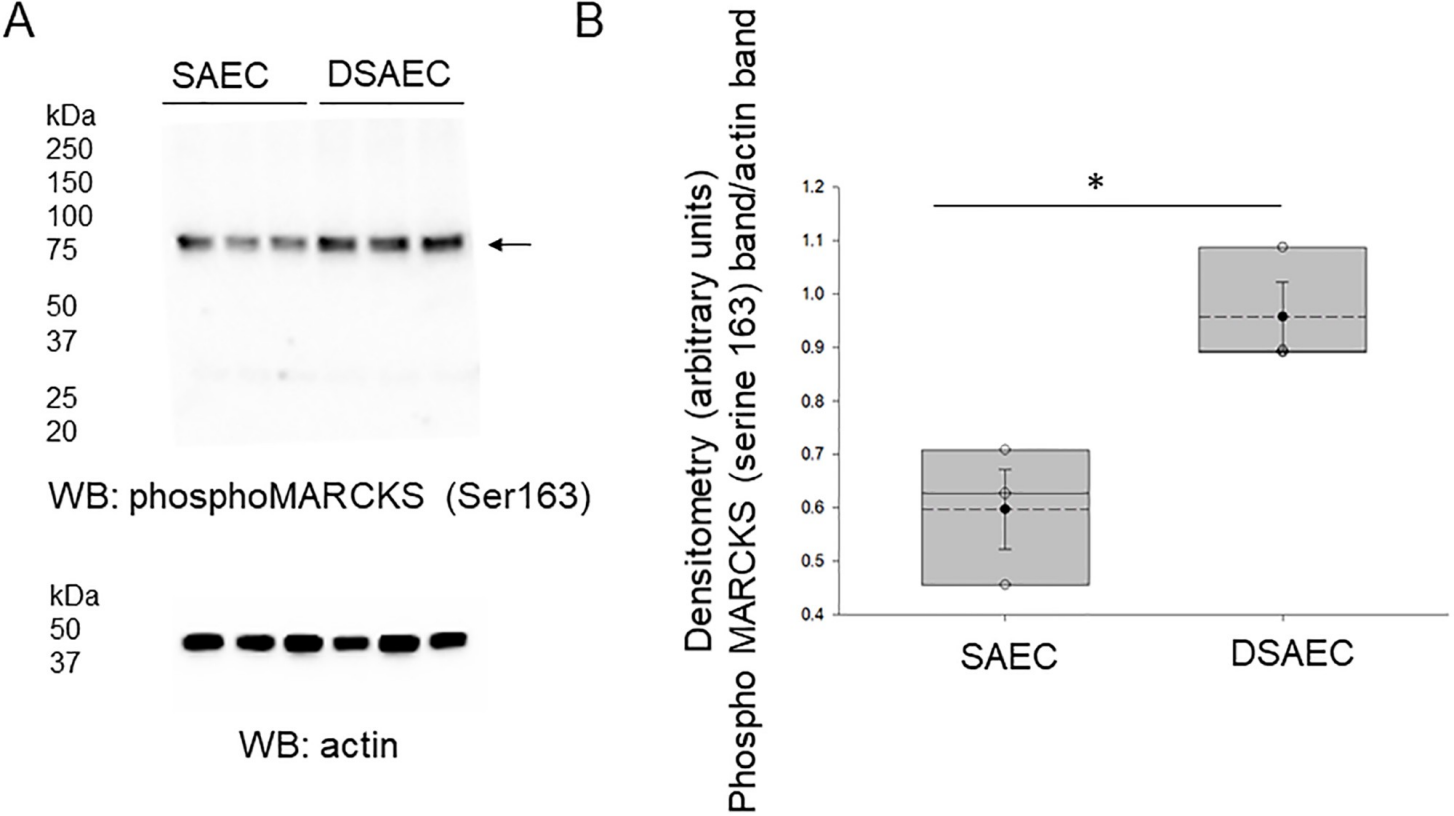
Western blot and densitometric analysis of phosphorylation of MARCKS at serine 163 in non-CF and CF cells infected with *M*. *avium*. A. Western blot analysis of MARCKS phospho serine 163 after treating CF and non-CF cells with *M*. *avium* (MOI 10) for 8 hours. B. Densitometric analysis of the MARCKS phospho-serine 163 band normalized to the actin band. * indicates a p-value of <0.05. SAEC refers to non-CF small airway epithelial cells and DAEC refers to CF small airway epithelial cells.

Since MARCKS is a prominent substrate of PKC which is shown by our data to be sensitive to *M*. *avium* infection, we also investigated whether infection with 10 MOI of *M*. *avium* for 8 hours would result in phosphorylation of serine 163 within the effector domain of MARCKS protein. CF and non-CF cells were lysed after *M*. *avium* infection, and the proteins were resolved by SDS-PAGE before probing for phospho-serine residue 163 by Western blotting. Western blot and densitometric analysis show CF cells have greater phosphorylation at serine residue 163 compared to non-CF cells after *M*. *avium* infection (Fig 8).

**Fig 9:**
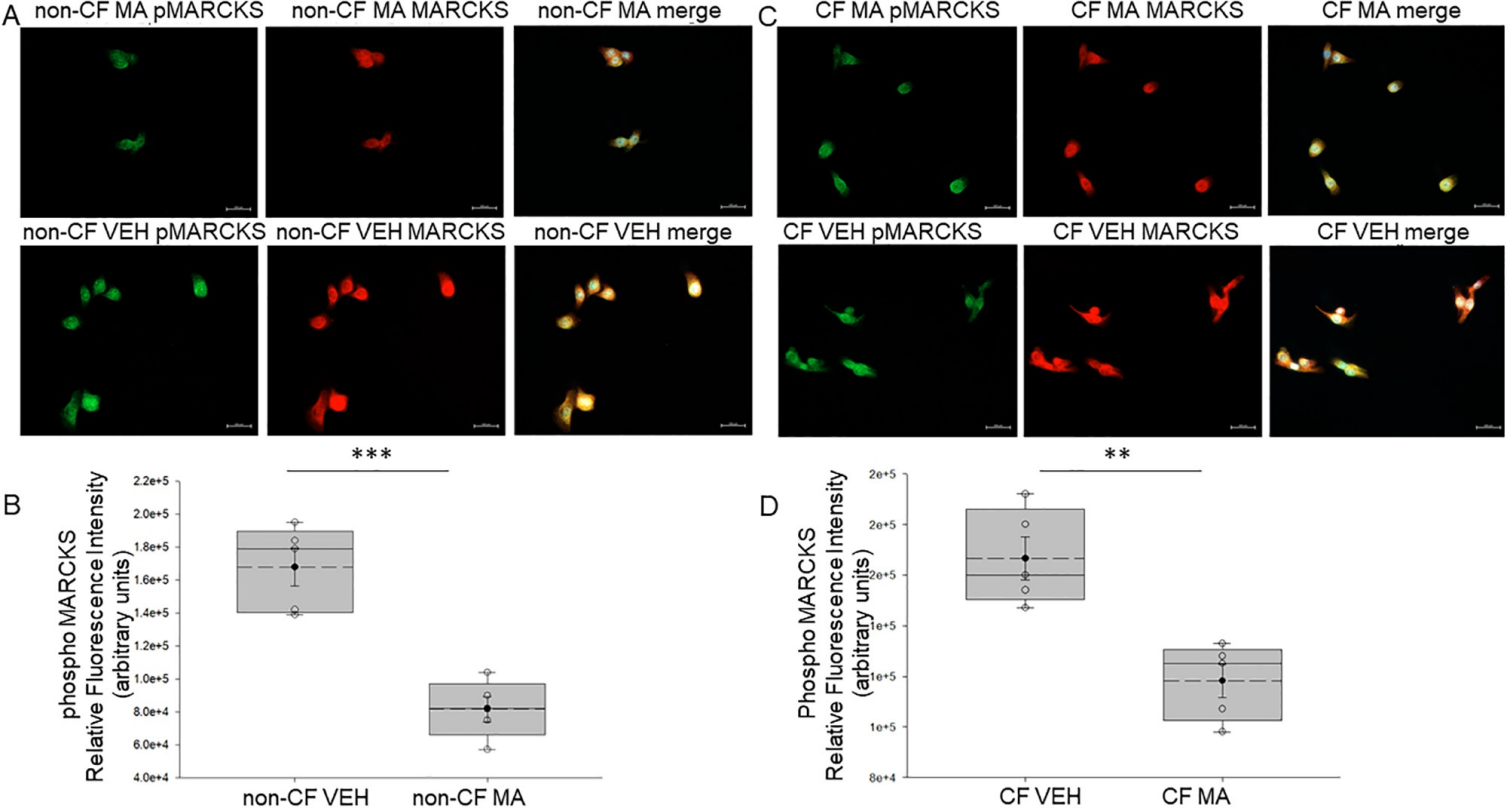
Immunofluorescence microscopy analysis of MARCKS phosphoserine 163 in non-CF and CF cells infected with *M*. *avium*. A. Images from non-CF and CF cells after treating the cells with vehicle or *M*. *avium* (10 MOI) for 8 hours. Scale bar is 500μm in each image. The merged image includes DAPI staining of the nuclei. B. Quantification of the fluorescence intensity of the images in panel A. VEH refers to vehicle and MA refers to SAEC refers to *M*. *avium*. SAEC refers to non-CF small airway epithelial cells and DAEC refers to CF small airway epithelial cells. Quantification of the fluorescence intensity was performed on 5 cells in a field for each group of cells. ** refers to a p-value <0.01. *** refers to a p-value <0.001.

Next, we aimed to investigate whether MARCKS phosphorylation at serine residue 163 in CF cells and in non-CF cells is affected by *M*. *avium* infection. Immunofluorescence microscopy studies showed that in both non-CF and CF cells, MARCKS phosphorylation at serine residue 163 was significantly attenuated after *M*. *avium* infection (Fig 9).

## Discussion

Here we show MARCKS protein expression and importantly how its phosphorylation is regulated by *M*. *avium* in non-CF and CF small airway epithelial cells. Mechanistically, we investigated the activity and expression of PKC and cathepsin B, respectively, in non-CF and CF cells, as these proteins are known to regulate MARCKS subcellular expression and function in other cell types. In this study, PKC activity (Fig 1) and cathepsin B protein expression (Fig 2) were found to be sensitive to *M*. *avium* infection in CF cells. Prior studies detected the presence of cathepsins B, L, and S in CF sputum [20, 36]. Differences in cathepsin levels between our study and other studies may be attributed to sampling differences, as our study investigated intracellular, rather than extracellular (i.e., sputum or BALF) cathepsin protein expression. Additionally, other variables, including age, have been shown to affect cathepsin concentrations in CF patients [37]. Laguna et al. showed the levels of urinary cathepsin B is significantly higher in CF infants compared to healthy infants [38]. Tan et al. showed that cathepsin B plays a role in elevated sodium resorption in CF airways [15]. Indeed, it has been shown that cathepsin secretion in the airways can occur secondary to certain cytokinetic stimuli, such as IL-13 and interferon (IFN)-gamma [37, 39].

One important and novel observation of this study is the demonstration of PKC activity significantly attenuated in both CF and non-CF cells infected with *M*. *avium*. This finding was surprising since a published study by Dechecchi et al. showed that PKC activates CFTR mediated chloride transport in cultured cells [32]. Another significant and novel finding from this study is that protein expression of cathepsin B was found to be augmented after infection of CF cells with *M*. *avium*. Rogan et al. found that *P*. *aeruginosa*-colonized CF cells had approximately threefold higher cathepsin expression than *P*. *aeruginosa*-negative cells and hypothesized that the antimicrobial glycoprotein lactoferrin was cleaved rapidly by cathepsins in the disease state of CF to prevent *Pseudomonal* biofilm formation [38]. Moreover, Vankayalapati et al. demonstrated that IFN-gamma, TNF-alpha, and IL-10 were produced by healthy alveolar macrophages exposed to *M*. *avium* complex [39]. It is possible that increased secretion of these cytokines by *M*. *avium* complex-infected SAEC stimulates cathepsin secretion and activation.

In the current study, our pathway analysis and proteolysis data determined that a myriad of biological processes are implicated upon NTM infection in CF and non-CF cells, most notably, the molecular function associated with the nucleus and cytoplasm, and kinase processes. In addition, our proteomics data (Fig 6) and protein biochemistry data (Fig 7) identified MARCKS protein as being upregulated in CF cells compared to non-CF cells. Moreover, our proteomics data showed MARCKS protein is sensitive to *M*. *avium*, as MARCKS is upregulated in CF cells infected with *M*. *avium* compared to non-CF cells infected with the microorganism. This observation is interesting for several reasons. First, in this study, we showed that PKC and cathepsin B are differentially expressed in CF and non-CF cells. MARCKS protein is regulated by both PKC activity and proteases including cathepsin B. PKC [40, 41] and Rho-associated protein kinase (ROCK) kinases [42] are known to phosphorylate the effector domain of MARCKS and regulate its subcellular localization. Proteases are thought to cleave MARCKS at sites upstream of its carboxy terminus tail and inhibit PKC from accessing and phosphorylating serine residues within the effector domain [43, 44]. Second, previous studies have shown there is hyperactive ENaC activity in CF cells [45]. Since MARCKS is known to stabilize ENaC at the plasma membrane and maintain the channel in an open conformation [40, 41, 44], upregulation of MARCKS may contribute to the upregulation of ENaC seen in CF.

Although this study presents several novel findings that could assist in elucidating the disease mechanism, there are some limitations. Primarily, only investigated *M*. *avium*, which may not be representative of NTM mechanisms as a whole. Additionally, while we have identified the relationship between NTM infection and the observed alterations in cathepsin levels and PKC downregulation within CF airway epithelial cells, we acknowledge that we did not focus on the relationship between cathepsins and PKC and can only speculate on the possible mechanism of PKC regulation by cathepsin as proposed by Kawakibi et al. [46]. Understanding the potential regulatory interactions between the PKC and cathepsin pathways could provide valuable insights into the broader molecular mechanisms driving these observed cellular responses. Future research endeavors should consider exploring this specific aspect to further enrich our understanding of the intricate molecular pathways involved in CF-related responses to NTM infection. Another limitation is that we did not investigate the molecular pathway disparities between NTM and typical mycobacterial species (TM) in the context of their interactions with CF airway epithelial cells. While our investigation has shed light on the distinct cellular responses elicited by NTM infection, we have not explored the intricate molecular nuances that may differentiate NTM from TM with regards to their influence on cellular pathways. Gaining a thorough understanding of these differential pathways could provide crucial information regarding the unique signaling pathways and mechanisms employed by NTM, allowing for more targeted therapeutic interventions. Subsequent research endeavors should consider addressing this gap in knowledge to unravel the complex molecular determinants that distinguish NTM from other mycobacterial counterparts. Both *Mycobacterium tuberculosis* (MTB), known for causing tuberculosis, and NTM are implicated in pulmonary diseases. However, MTB mechanisms have been more extensively researched [47]. The biological pathways of TB and NTM diseases share certain fundamental events contributing to the bacterial intracellular survival advantage, such as apoptosis induction strategies, however, striking differences, such as host-susceptibility, exist [47].

We previously showed the subcellular localization and function of MARCKS protein is regulated by proteolysis in renal epithelial cells. Renal MARCKS protein is a substrate of proteases including cathepsins and calpains. Phosphorylation of serine residues within the basic effector domain of renal MARCKS protein is mediated by PKC and is thought to cause translocation of the protein to the cytoplasm. Proteolysis at sites after the effector domain of renal MARCKS is thought to prevent PKC from accessing and phosphorylating serine residues within the effector domain. The data from this study shows for the first time that CF cells have greater phosphorylation at serine 163 compared to non-CF cells after *M*. *avium* infection (Figs 8 and 9). The data from this study shows protein expression of cathepsin B is augmented in CF cells compared to non-CF cells infected with *M*. *avium* (Fig 2). Since there was both an increase in phosphorylation at serine 163 of MARCKS protein and an increase in expression of cathepsins B, the mechanism for the subcellular localization and function of MARCKS in small airway epithelial cells is likely different compared to in renal epithelial cells. Presumably, there are other post-translational modifications of MARCKS that are responsible for regulating its function in small airway epithelial cells. Additional studies warrant further investigation of MARCKS regulation in CF and non-CF cells under both basal conditions and after infection of *M*. *avium*.

**Fig 10:**
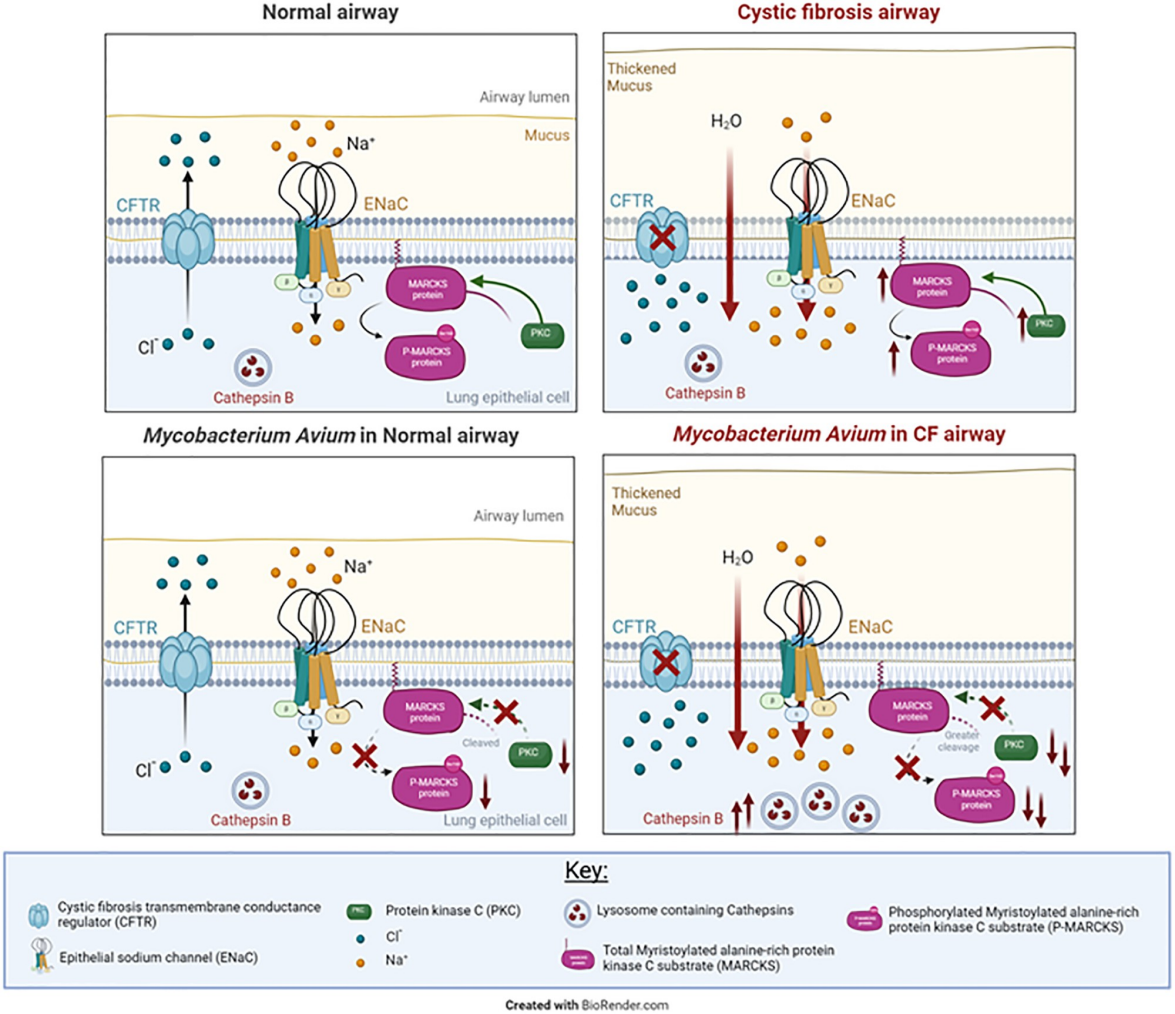
Proposed model showing the regulation of cathepsins and MARCKS in CF and non-CF cells by *M*. *avium*. CF and non-CF cells infected with *M*. *avium* have decreased protein kinase C (PKC) activity and increased protein expression of cathepsin S and B. Both PKC and cathepsins are known to regulate the myristoylated alanine-rich C-kinase substrate (MARCKS) protein. Proteomic analysis validated by Western blot analysis showed MARKCS protein expression is augmented in CF cells compared to non-CF cells and augmented in cells infected with *M*. *avium*.

Our proposed schema (Fig 10) illustrates the outcomes of our investigation, identifying elevated cathepsin B levels within NTM-infected CF airway epithelial cells, concomitant with the observed downregulation of protein kinase C (PKC). These findings collectively emphasize the intricate interplay between NTM infection, cathepsin B abundance, and the modulation of PKC-signaling pathways in CF-associated cellular contexts.

## Supporting information

S1 Graphical abstract (TIF)
