# Supplementary figures and images for: Mycobacterium avium inhibits protein kinase C and MARCKS phosphorylation in human cystic fibrosis and non-cystic fibrosis cells
Source: PLoS One. 2024 Oct 16;19(10):e0308299. doi: 10.1371/journal.pone.0308299 (PMC11482691; doi:10.1371/journal.pone.0308299)

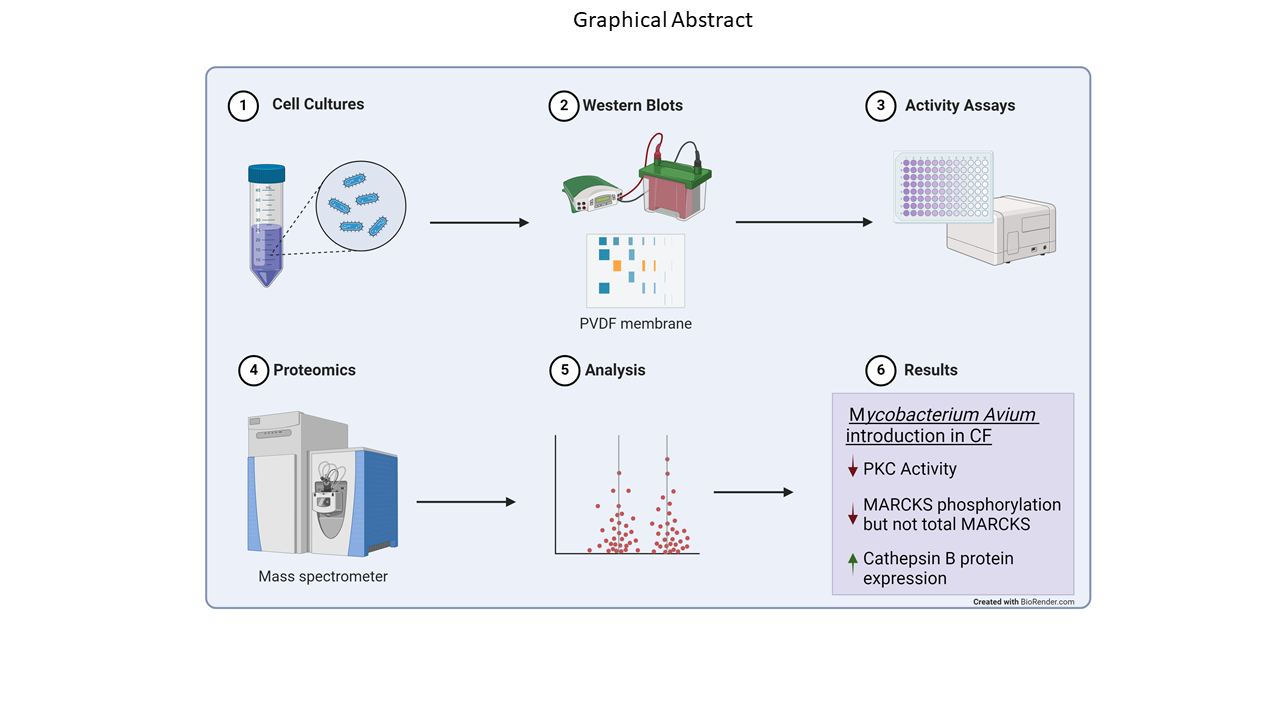

Supplement: S1 Graphical abstract — (TIF) [file pone.0308299.s001.tif]
